# Editorial: Novel Actuators, Sensors and Control Systems for Endoscopic Robots

**DOI:** 10.3389/frobt.2021.797467

**Published:** 2021-11-22

**Authors:** Luigi Manfredi, Leonardo S. Mattos, Andreas Melzer

**Affiliations:** ^1^ Institute for Medical Science and Technology, IMSaT, University Dundee, Dundee, United Kingdom; ^2^ Advanced Robotics Department, Istituto Italiano di Tecnologia, Genova, Italy; ^3^ Institute and Innovation Center for Computer Assisted Surgery, University Leipzig, Leipzig, Germany

**Keywords:** endoscopic robots, endorobots, laser microsurgery, image robotics, soft robotics

## Introduction

Design of medical robots should arise from a close collaboration between clinicians and scientists and as a result of the identification of unmet clinical needs. Starting from this early stage, where an idea is just conceived, concept designs and proof of concepts are the following steps. This is a long journey that eventually may terminate with a product that is available at clinical stage to improve the health care quality for patients. Through this journey, an important role is played by the selection of the appropriate components to be used for the development of the device. Actuators, sensors, and the way to control these components can decide the success or unsuccess of an idea. Precision, safety, reliability, and dexterity in narrow areas are some of the aspects that have spread the use of medical robots in current medical practice. To gain access to difficult-to-reach organs, endoscopic robots or endorobots, need to be small, flexible, smart, and versatile. The main challenge in designing an endorobot stands in the limited space that surrounds the device, which restricts the use of standard and off-the shelf components (Manfredi et al., 2017) (Manfredi and Cuschieri 2018). To gain access through small apertures or natural orifices, scientists need to provide innovative solutions as well as produce and assemble these prototypes “in-house”. Soft materials are nowadays used as an alternative paradigm to design miniaturised actuators (Manfredi et al., 2018a) (Manfredi et al., 2018b) (Manfredi et al., 2019a) (Manfredi and Cuschieri, 2019) (Manfredi et al., 2019b) and sensors. These materials are soft with variable geometries. Compliance remains the main advantage, presenting mechanical properties similar to humans’ organs. However, precision, and low output force or torque at small scale are still limitations to be taken into account.

## Contents of the Research Topic

This research topic includes a collection of articles and reviews that can be summarised in three main themes: 1) GI (gastrointestinal) endoscopy, 2) laser microsurgery, and 3) imaging robotics.

### GI Endoscopy

Endorobots can have various medical applications. For example, inspection of the GI tract is performed by using an endoscope, which consists of a long tube, most of which has a passive mechanical system that relies on forces exchanged with the GI wall to bend and follow the GI shape. These forces cause pain, discomfort and can damage the mucosa or even cause perforation. Several companies have tried to produce, certify, and commercialise medical devices for colonoscopy. Research institutes are working on novel concepts that reduce pain and discomfort, facilitate the procedure, are cost-effective, and single use to avoid disease cross-contamination (Manfredi). Soft actuators are an alternative solution to the rigid counterparts, because they can reduce forces applied to the GI wall and potentially improve the acceptability of the procedure. An origami-based soft robotic actuator for upper GI endoscopic application was presented in (Chauhan et al.). The proposed origami design was constructed and tested. Experiments showed flexibility in bending and permitted continuous change in the bending plane, three actuations and one channel for cabling a camera, compact fabrication, and production of an actuator without damage in an elastomeric material. Manoeuvrability of soft and continuous devices still face many open challenges in modelling and control, especially when a task requires a closed-loop controller (Isbister et al.). developed an analytical model and then validated it through experiments by using an endoscopic continuum robot. The experimental setup consisted of a system actuated by four tendons via a motorised lead screw mechanism with load cells. The deformation of the backbone was measured using a contactless optical tracking system. When the model was unloaded, the prediction was very similar to the deformations of the backbone.

### Laser Microsurgery

When the requirements in precision become high in delicate organs, such as larynx, laser microsurgery is one of the currently well accepted techniques. The European project μRALP has pioneered research to redesign the current laser microsurgery approach, transforming it into an endoscopic surgery procedure (Mattos et al.). They have developed robotic micro-technologies to enable the miniaturization of the surgical system and produce an advance device that includes hardware and software infrastructure for remote operation, accurate and robust laser visual servoing for 3D control of the laser spot in unknown environments, and augmented reality to plan and control laser incisions in 3D.

### Imaging Robotics

Imaging robotics to develop novel assistance systems has been developed since 2000 (Unger et al.). reports a review on the latest advances in academia and applied research on commercial devices. The high precision and reproducibility of robot-assistance enable a wide range of applications. This review covers a comprehensive description on computer tomography (CT) guided interventions, magnet resonance imaging (MRI), robotic systems for MR-guided interventions and robotic assisted US-guidance (Patel et al.). presents a body mounted robot for MRI-guided surgery, designed to be compact and lightweight, constructed with nonmagnetic material for MRI safety. The authors aim to move away from the current two-step arthrography procedure, which consists of a CT/x-ray guided needle insertion followed by diagnostic MRI, into a single-step ionising radiation-free procedure under MRI guidance. The robot was evaluated on a Thiel soft embalmed cadaver, attached to the shoulder using straps. The experiments showed that the needle was successfully placed in the shoulder joint space in all the targeting attempts with high accuracy, and no image quality degradation was detected due to the robot.

## Conclusion

The manuscripts published in this research topic show recent advancements in medical robotics with particular attention to endoscopy (actuators and control strategies of continuum robots), laser microsurgery, and image guidance. The wider adoption of medical robots in health centres, and the recent commercialisation of new robotics devices, have increased their acceptability in health care centres and patients. More effort is still required to widen the clinical application areas and to reduce the impact of cost for health care centres in adopting new technologies.
